# Rhinorrhée cérébro-spinale post traumatique tardive dévoilant une tumeur cérébrale

**DOI:** 10.11604/pamj.2015.22.242.8099

**Published:** 2015-11-13

**Authors:** Rim Lahiani, Madiha Mahfoudhi

**Affiliations:** 1 Service ORL, Hôpital Charles Nicolle, Tunis, Tunisie; 2 Service de Médecine Interne A, Hôpital Charles Nicolle, Tunis, Tunisie

**Keywords:** Brèche méningée, rhinorrhée cérébrospinale, tumeur cérébrale, meningeal breach, cerebrospinal rhinorrhea, brain tumor

## Image en medicine

Une brèche ostéo-durale post-traumatique peut se révéler de façon tardive, à l'occasion d'une augmentation de la pression intracrânienne. La majorité des rhinorrhées cérébrospinales post traumatiques s'estompent spontanément. La cicatrisation méningée peut être imparfaite. Une hyperpression du Liquide céphalorachidien à son contact peut faire reparaitre la rhinorrhée. De ce fait, toute fuite post traumatique tardive de liquide céphalo-rachidien doit faire rechercher des facteurs de risque d'hypertension intracrânienne. Le traitement est alors chirurgical pour prévenir le risque de méningite. Un homme âgé de 26 ans, aux antécédents de traumatisme crânien avec perte de connaissance initiale il y a 3 ans (TDM cérébrale initiale sans anomalies), a consulté pour une rhinorrhée claire unilatérale droite évoluant depuis 06 mois, récidivante et aggravée par la position proclive. L'examen physique a révélé une rhinorrhée claire droite d'aspect eau de roche et de faible abondance sans notion de fièvre ni de syndrome méningé. L'examen neurologique était normal. L'étude du liquide de rhinorrhée a conclut au liquide céphalo-rachidien. L'IRM cérébrale a objectivé une méningo-encéphalocèle frontale droite, un processus intra-ventriculaire du troisième ventricule de 23 x 20 x 24cm avec hydrocéphalie en amont non active évoquant une tumeur gliale de bas grade. La TDM du massif facial a révélé une brèche osseuse de 1 cm du toit du sinus frontal droit avec présence de méningocèle. Le patient a été opéré en neurochirurgie par ventriculocisternestomie endoscopique et a bénéficié d'une réfection de la brèche ostéo-durale par volet frontal. Les suites opératoires étaient simples.

**Figure 1:**
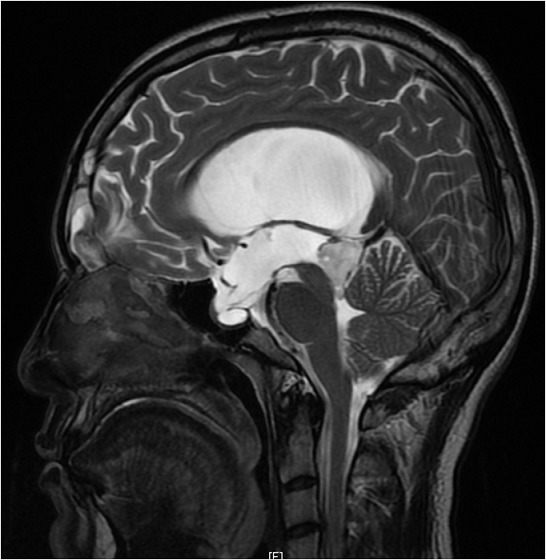
IRM cérébrale (coupe sagittale): processus intra-ventriculaire du troisième ventricule en hypersignal hétérogène en T2

